# The Acrocallosal Syndrome in A Neonate With Further Widening of Phenotypic Expression

**Published:** 2014

**Authors:** Ravish SINGHAL, Sadbhavna PANDIT, Ashok SAINI, Paramjit SINGH, Neeraj DHAWAN

**Affiliations:** 1 PG resident, Department of Pediatrics, Government Multispecialty Hospital, sector-16, Chandigarh, India; 2 Head of the Department Pediatrics, Government Multispeciality Hospital, Sector-16, Chandigarh, India; 3 Medical Officer, Pediatrics, Government Multispecialty Hospital, sector-16, Chandigarh, India

**Keywords:** Acrocallosal syndrome (ACLS), Agenesis of corpus callosum, Polydactyly

## Abstract

The presentation of the typical characteristics of the acrocallosal syndrome (ACLS) are hypoplasia/agenesis of corpus callosum, moderate to severe mental retardation, characteristic craniofacial abnormalities, distinctive digital malformation, and growth retardation in a neonate.

An Indian neonate presented on day 1 of life (youngest in the literature to be reported) with combination of abnormalities consistent with the acrocallosal syndrome and some additional findings. The baby, born to non-consanguineous, healthy parents, presented with macrocephaly, prominent forehead, hypertelorism, polydactyly of the hands and feet, duplication of hallux, hypotonia, recurrent cyanotic episodes, rib anomalies, dextro-positioning of heart, and delayed fall of umbilical cord.

As the mode of inheritance of ACLS is autosomal recessive, the risk of recurrence is 25%. Genetic counselling is of prime importance, Polydactyly, and central nervous system malformations can be detected by ultrasonography in the second trimester, but due to variability of presentation, prenatal diagnosis may not always be possible.

## Introduction

The acrocallosal syndrome (ACLS) was first described by Schinzel (1). ACLS is a rare genetic disorder. The few typical characteristics of ACLS are hypoplasia/ agenesis of corpus callosum, moderate to severe mental retardation, characteristic craniofacial abnormalities, distinctive digital malformation, and growth retardation.

However, symptoms and physical findings in ACLS may be quite variable. Although autosomal recessive inheritance has been suggested, ACLS often appears to occur sporadically (2).

## Case report

A full term female vigorous newborn, delivered vaginally with meconium stained liquor as a result of non-consanguineous marriage (maternal age 25 years and paternal age 28 years) presented to us on day 1 of life with a history of not sucking well, respiratory difficulty, and recurrent cyanosis. She was the product of third pregnancy, the first being a spontaneous abortion at 8 weeks of gestation and second being a healthy term male baby. Birth weight was 3.2 kg (50th centile) and length was 48 cm (10th-25th centile) with a head circumference of 36cm (50^th^ 97th centile). Antenatal period was uneventful and there was no history of exposure to any known or probable teratogens during pregnancy. No other case of birth defect or mental retardation was known in the family.

**Fig 1:**
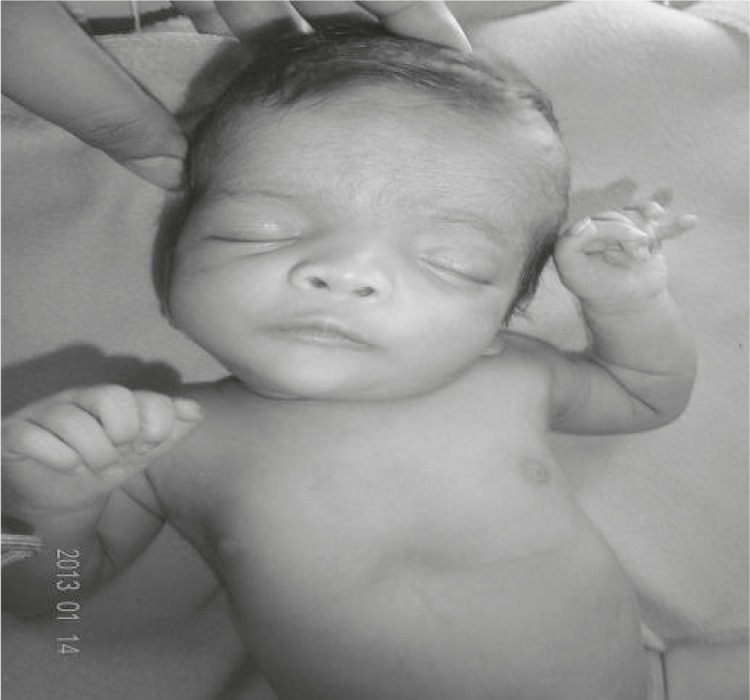
Downslanting & hyperteloric eyes

**Fig 2:**
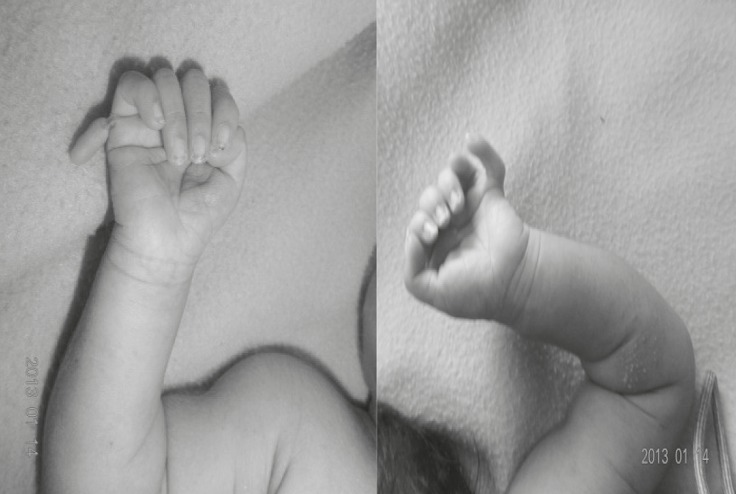
Right & left hand post axial polydactyly

**Fig 3:**
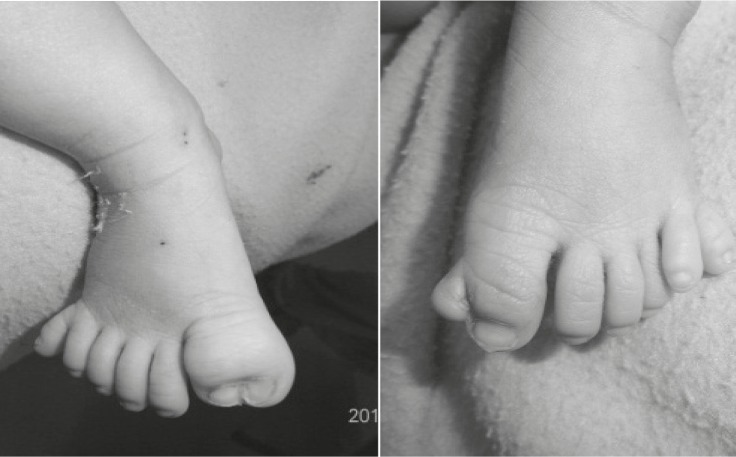
Duplicated hallus with post axial polydactyly

On physical examination, the baby had dysmorphic facial appearance with prominent forehead, hyperteloric eyes (inner canthal distance of 2.6cm and outer canthal distance of 8.6cm, both> +3SD), flat nasal bridge, downward slanting eyes, low set ears, enlarged philtrum, and widely spaced nipples (figures 1). There was postaxial polydactyly of both hands and both feet as well as duplication of both hallux (figures 2 and 3). 

A neurological examination revealed generalized mild hypotonia, sluggish neonatal reflexes with normal deep tendon reflexes, and no history or evidence of seizures. 

**Fig 4:**
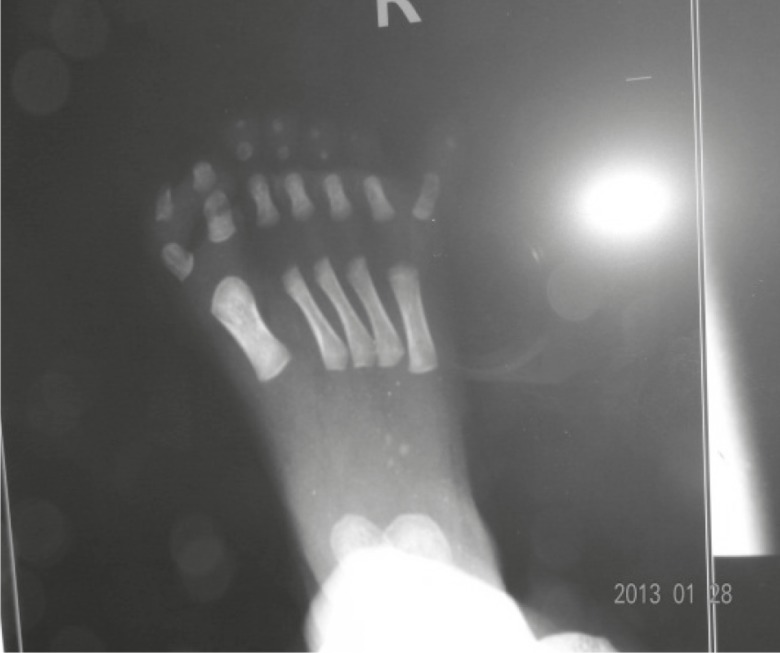
Right foot with seven phalanges polydactyly

**Fig 5:**
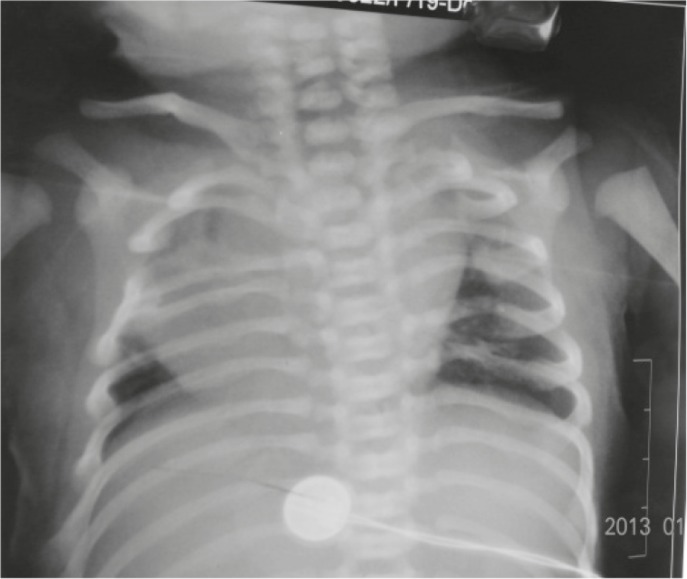
Widened right 2nd intercoastal space

**Fig 6:**
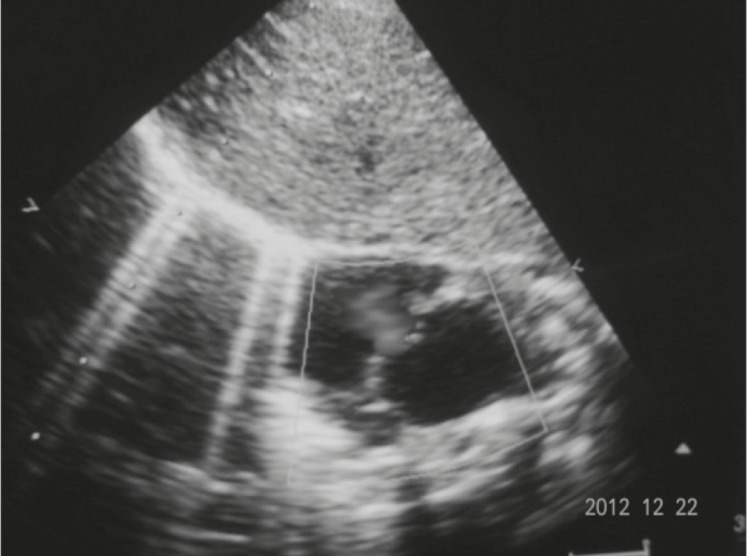
ECHO showing ASD left to right shunt

On chest examination, the baby had tachypnea with no adventitious sounds. Cardiovascular examination showed maximum cardiac impulse predominantly on the right side with short ejection systolic murmur in the right 4th parasternal space. The baby had a grossly delayed fall of umbilical cord (24th day), otherwise abdominal and genital examination were unremarkable. An x-ray showed supernumerary phalanges in both hands and feet and duplication of phalanges of the halluces bilaterally (figure 4). A chest x-ray showed heart shadow predominantly on right side with widening of 2nd and narrowing of 3rd right intercostal space (figure 5). Echocardiography (ECHO) revealed small secundum type Atrial Septal Defect (ASD) (figure 6) with moderate tricuspid regurgitation and dextro positioning of the heart. 

**Fig 7:**
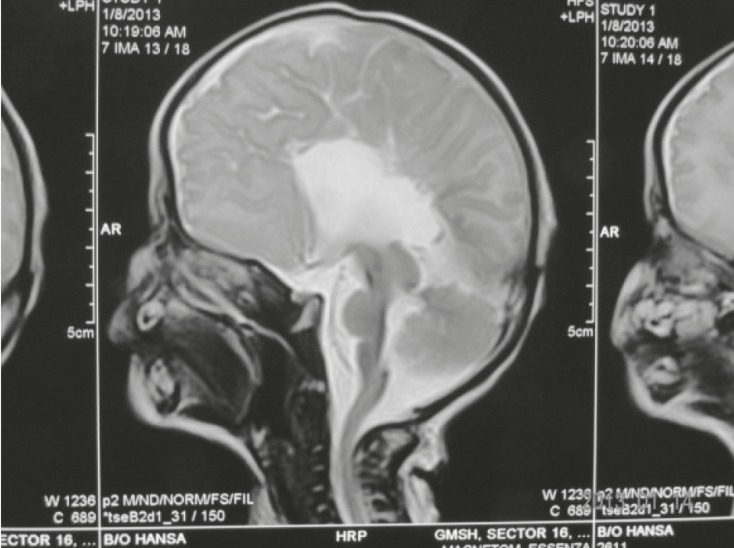
Coronal MRI showing corpus callosum Agenesis

**Fig 8:**
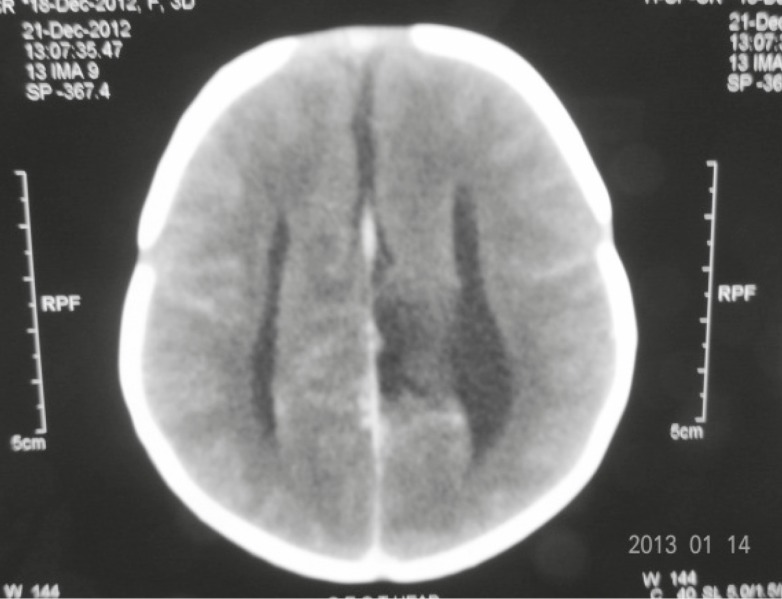
Axial CT scan showing parallel ventricles

A cranial Computed Tomography scan (CT scan) and Magnetic Resonance Imaging (MRI) revealed corpus callosum agenesis and parallel and prominent lateral ventricles with high positioned third ventricle (figures 7 and 8). The chest CT scan revealed dextro positioning of the heart. An eye examination was normal and there was no clinical evidence of deafness. USG abdomen and pelvis was normal. Two septic screens were done and were negative. The metabolic and thyroid profile was normal. The karyotype report was normal.

**Table 1:** Features of Acrocallosal Syndrome

**No.**	**Features**	**Authors**	**In our case**
1	Macrocephaly	Schinzel and Schmid(1980) (3)	+
2	Agenesis/hypoplasia of corpus callosum	Moeachler et al (1987) (4)	+
3	Seizures/abnormal EEG	Schinzel et al(1986) (5), Koenig et al (2002) (6)	-
4	Hypertelorism and frontal bossing	Schinzel(1988) (7)	+
5	Optic atrophy	Smith (8)	-
6	Hallux duplication	Schinzel and Schmid(1980) (3)	+
7	Pre and Post axial Polydactyly of toes and fingers	Schinzel (1988) (7), Smith (8)	+
8	Cerebellar hypoplasia	Hendrik et al (1990) (8)	-
9	Cyanotic spells	Yuksel M et al (1990) (9)	+
10	Hypotonia	Yuksel M et al (1990) (9)	+
11	Congenital heart disease	Casamassima et al (1989) (10)	+
12	Dextro position of heart	None	+
13	Delayed fall of cord	None	+
14	Rib anomalies	None	+

+ present – absent

## Discussion

Acrocallosal syndrome is a rare genetic disorder first described by Schizel in a four-year-old boy. The pattern of multiple congenital anomalies found in ACLS include typical facial features, agenesis of corpus callosum, bilateral duplication of halluces, duplication of thumb, umbilical hernia, hypotonia, seizures, and severe motor and mental retardation. In view of the clinical variability and the fact that facial dysmorphism is not always characteristic, the diagnosis of ACLS may sometimes be difficult and subject to debate. Courtens et al (1997) laid down the minimal diagnostic criteria for this condition (11). These are: 1. Total or partial absence of corpus callosum; 2. Minor craniofacial anomalies (prominent forehead, hypertelorism, short nose with anterverted nostrils, large anterior fontanel; 3. Moderate to severe psychomotor retardation (with hypotonia); and 4. Polydactyly. The presence of 3 out of 4 criteria together with other associated findings could lead one to suspect the diagnosis of ACLS. Our case had all the features typical of ACLS. Phenotypic expression of ACLS can be further widened with inclusion of dextroposition of heart, rib anomalies, and delayed fall of umbilical cord (Table 1) as these have not been reported in the literature to date. 

There are other conditions with midline abnormalities and Polydactyly or other digital anomalies. These include Greig cephalopolysyndactyly, oral facialdigital syndrome type 2, Smith-Lemli-Opitz syndrome, and Rubenstein Tyabi syndrome. These conditions can be excluded as they usually exhibit other characteristic features, which allow easy differentiation from ACLS. 

As the mode of inheritance of ACLS is autosomal recessive, the risk of recurrence is 25%. Genetic counselling is of prime importance, Polydactyly, and central nervous system malformations can be detected by ultrasonography in the second trimester, but due to variability of presentation, prenatal diagnosis may not always be possible. The homozygous p.N1060S missense mutation in a highly conserved residue in KIF7(15q26.1), a regulator of ciliary Hedgehog signaling that has been recently found to be the cause of Joubert syndrome, fetal hydrolethalus, and acrocallosal syndromes(12). The mutation most likely influences the early development of midline structures during embryogenesis.

## Conflict of interest

The authors declare no conflict of interest.

## Author contributions

Dr Ravish Singhal: Found the case, doing complete examination & diagnosis Dr Sadhbavna Pandit, Ashok Saini, Paramjit Singh, Neeraj Dhawan: Critical review of the case
